# Management of alcohol use disorder in patients with chronic liver disease

**DOI:** 10.1097/HC9.0000000000000145

**Published:** 2023-06-14

**Authors:** Jessica L. Mellinger, Anne C. Fernandez, G. Scott Winder

**Affiliations:** 1 Division of Gastroenterology and Hepatology, Department of Internal Medicine, Michigan Medicine, Ann Arbor, Michigan, USA; 2 Department of Psychiatry, Michigan Medicine, Ann Arbor, Michigan, USA; 3 Department of Surgery, Michigan Medicine, Ann Arbor, Michigan, USA; 4 Department of Neurology, Michigan Medicine, Ann Arbor, Michigan, USA

## Abstract

Alcohol use disorder (AUD) rates have risen dramatically in the United States, resulting in increasing rates of alcohol-associated liver disease (ALD), but many patients struggle to access alcohol use treatment. AUD treatment improves outcomes, including mortality, and represents the most urgent means by which care can be improved for those with liver disease (including ALD and others) and AUD. AUD care for those with liver disease involves 3 steps: detecting alcohol use, diagnosing AUD, and directing patients to alcohol treatment. Detecting alcohol use can involve questioning during the clinical interview, the use of standardized alcohol use surveys, and alcohol biomarkers. Identifying and diagnosing AUD are interview-based processes that should ideally be performed by a trained addiction professional, but nonaddiction clinicians can use surveys to determine the severity of hazardous drinking. Referral to formal AUD treatment should be made, especially where more severe AUD is suspected or identified. Therapeutic modalities are numerous and include different forms of one-on-one psychotherapy, such as motivational enhancement therapy or cognitive behavior therapy, group therapy, community mutual aid societies (such as Alcoholics Anonymous), inpatient addiction treatment, and relapse prevention medications. Finally, integrated care approaches that build strong relationships between addiction professionals and hepatologists or medical providers caring for those with liver disease are crucial to improving care for this population.

## INTRODUCTION

Alcohol use disorder (AUD) and alcohol-associated liver disease (ALD) rates have risen dramatically in the United States. Alcohol cessation is the most important aspect of long-term survival for those with ALD, but hepatology providers often find managing alcohol use challenging. In this review, we will examine the changing epidemiology of ALD, including the increases among women and young people. Then, we will present the best ways to detect alcohol use, determine how severe an alcohol use problem your patient has, and review the types of AUD treatment and ways to motivate your patient with ALD to engage in treatment and reduce or stop alcohol use.

## EPIDEMIOLOGY

The United States has seen, in recent years, a dramatic increase in ALD rates.^1^ In the United States, the prevalence of all stages of ALD has been estimated at 8%, but the proportion with cirrhosis has grown from 2.2% to 6.6% from 2001 to 2016.^2^ Over a 16-year period, mortality for all-cause cirrhosis rose 65%, but these increases were almost entirely due to rises in ALD mortality, especially among young people ages 25–34 and women.^3^ Globally, increases in both compensated and decompensated alcohol–associated cirrhosis have also been demonstrated, rising from 288 per 100,000 persons to 290 per 100,000 persons from 1990 to 2017 for compensated cirrhosis and 24.3 per 100,000 persons to 30 per 100,000 persons for decompensated cirrhosis over the same timeframe.^4^

### Mortality

As a result, ALD is now the greatest contributor to liver-related mortality in Europe, making up 40%–50% of total liver disease deaths attributed to ALD (though rates vary from country to country).^4^ A biopsy-based study of long-term mortality in a cohort of Swedish patients with ALD followed showed an overall 5-year mortality for all stages of ALD of 40.9% versus 5.8% in the non-ALD reference population.^5^ However, even for early-stage alcohol–associated steatosis, mortality was 5.2% versus 0.5% in matched controls, with a higher risk for mortality in those with more advanced ALD (adjusted HR: 6.07, 95% CI, 5.43–6.77 for cirrhosis; adjusted HR: 2.72, 95% CI, 2.2–3.35 for those with steatosis only).^5^ These dramatic increases in ALD have been amplified and accelerated by the COVID-19 global pandemic, which saw rises in ALD hospitalizations, as well as waitlisting and transplants for acute alcohol-associated hepatitis (AAH) in the United States.^6–8^

### What is driving the increases in ALD?: Risk factors

Although various different factors influence the impact of alcohol use on the development and worsening of liver disease (eg, genetics, sex, comorbid liver disease, and obesity^9^), the overall dose of alcohol use over time remains the leading causative factor for ALD and increased mortality.^10–12^ For those with AAH, any consumption of alcohol 6 months following an index AAH hospitalization resulted in increased mortality of at least double for 1–2 drinks per day, increasing up to 6-fold for those consuming 100-g alcohol or more per day.^13^ Alcohol consumption in those with known cirrhosis increases the risk of first-time ascites, variceal bleeding, and mortality as well.^14,15^ In addition, while projected increases in obesity and NASH prevalence in the United States will increase liver disease and transplant burden,^16^ alcohol use in the obese population with or without metabolic syndrome has also been shown to increase rates of liver disease, including decompensation and mortality.^17–20^ As such, the combined effect of rising obesity and metabolic syndrome rates coupled with rising alcohol consumption in the United States may explain part of the increase in the ALD burden.

### Sex differences

In a meta-analysis of studies evaluating alcohol dose and risk of cirrhosis, there were clear sex disparities with women consistently more likely to get cirrhosis at lower drinking levels than men.^12^ Although as little as a single drink per day (compared with long-term abstainers) was associated with an increased risk of cirrhosis in women [relative risk (RR): 1.40, 95% CI, 1.00–1.97], 5–6 drinks per day were associated with an increased risk in both men and women (pooled RR: 6.26, 95% CI, 2.38–16.50).^12^ However, not all who drink heavily will go on to develop more advanced ALD, cirrhosis, or AAH. Chronic alcohol use of 20- to 50-g alcohol (~2–5 standard US drinks per day at 14-g alcohol per drink) in women and 60- to 80-g alcohol per day, sometimes termed “heavy drinking” (consuming 4 or more drinks per day for women and 5 or more drinks per day for men^21^) produces hepatic steatosis (or fatty liver) in 90% or more of drinkers.^22,23^ Certain drinking patterns have also been identified, which may be more damaging, especially for women. In the Million Woman Study in the United Kingdom, for example, while an overall increased dose of alcohol was associated with an increased risk of cirrhosis, certain drinking patterns, such as drinking with meals, were associated with a lower incidence of cirrhosis (RR: 0.69, 95% CI, 0.62–0.77).^24^

**FIGURE 1:**
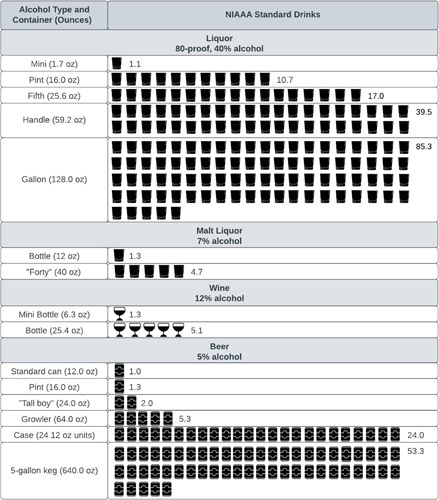
Number of standard drinks in commonly sold alcohol container volumes. Abbreviations: NIAAA, National Institute on Alcohol Abuse and Alcoholism.

Unsurprisingly, the dismal epidemiologic trends of increasing ALD have been preceded by similar rises in drinking across nearly all demographics, including all age ranges, races and ethnicities, and both sexes. Although global definitions of moderate drinking differ by country, the National Institutes of Health in the United States defines moderate drinking as anything over 1 drink per day for women or 2 drinks per day for men (Figure 1) although there is increasing suggestion that even this level of alcohol consumption may be unhealthy.^25^ In the United States, rates of alcohol use have risen across all demographics over the past 15 years though the most pronounced increases have been among women and young people.^26,27^ In a meta-analysis of several large US databases on alcohol use, past-year alcohol use and binge drinking rates increased for all demographics, with rates increasing most for women.^28^ In fact, the overall rate of drinking for men remained constant, while nearly the entirety of the increase in overall population rates was provided by increasing rates among women. Reasons for this rise in drinking among women are complex, but may be related to social and cultural factors, and advertising that targets women specifically, associating alcohol use with specific female life experiences (“wine moms” and “mommy juice”) and with friendship, camaraderie, and emotional support.^29,30^

## DETECTING ALCOHOL USE

Given these considerations, how should we approach alcohol use management in our patients with chronic liver disease? The first step is to find out if your patient is drinking. Detecting alcohol use can be done in 3 primary ways: elicitation during clinical history, using validated surveys, and through alcohol biomarkers.

### Quantifying alcohol intake by history

Querying patients about their alcohol use during the history is valuable but comes with some pitfalls. Asking patients how many drinks they have in a day may give an inaccurate answer, even in settings where hepatologists are asking about alcohol use regularly, such as transplant clinics.^31^ Knowledge of “what’s in a drink” may not be uniform across the population. In several studies of drink estimation, the number of standard drinks actually poured (when instructed to pour only 1 drink) ranged from 1.4 to 2.1 drinks.^32^ In addition, misconceptions about the harms of different types of alcohol to the liver are common, including that beer is not harmful to the liver, only hard liquor.^33^ As a result of these misconceptions, patients may underestimate or not disclose their alcohol use or erroneously switch beverages (liquor to beer), thinking that they are eliminating their risk. Alternate styles of questioning may be helpful in overcoming these challenges. For example, instead of asking “how many drinks do you drink in a day?”, asking what the patient drinks and then querying how long it takes him or her to get through a bottle of their drink of choice (eg, a bottle of wine or a fifth of vodka) may produce a more accurate assessment of total dose of alcohol consumed. See Table 1 for ways to approach counseling around alcohol use. Clinicians should be aware of how many standard drinks are in typical bottles and beverage containers to convey this information to patients who may be less aware of the total dose of alcohol that they are truly consuming (Figure 1).

**TABLE 1:** Challenges and potential approaches to discussing alcohol and substance use with patients

Challenges in discussing alcohol and substance use	Potential approaches
Inaccurate or imprecise patient estimates of alcohol use	Use a visual chart (Figure 2) to help patients understand how much alcohol is in a single drink Ask what patients drink and how long it takes them to get through the unit of alcohol the commonly purchase [eg, “how long it takes to get through a “fifth” (750 mL) of liquor,” or “how often would you purchase another “pint” (375 mL) because you ran out?”]
Stigma and shame	Use first-person plural pronouns to discuss substance matters to reduce confrontational undertones (eg, we, us) Normalize asking about alcohol use (eg, “Doctors commonly ask all their patients about we are doing with alcohol use,” “It is common for all of us to find it difficult to talk about our drinking with our doctor”) Use destigmatizing language to reduce defensiveness and encourage psychological openness (eg, instead of “addict” “substance abuse,” or “alcohol-associated,” say “person with alcohol use disorder” or “patients with alcohol challenges”; use substance use disorder or alcohol use disorder in general)^a^ Normalize AUD care and treatment as an expected part of medical and liver care and of equal importance and relevance. Project reasonable, proportional, and authentic optimism about patients’ potential for improvement
Ambivalence about changing alcohol use behaviors	Use a patient-centered, motivational interviewing ^34^ approach based on the following interview principles (use your **OARS**): **O**pen-ended questions: for example, “What brings you here today? How do you hope I might be able to help you today?” **A**ffirming: Center on the patient and their successes no matter how small (“you” not “I” language). For example, “You did a really good job of keeping track of your drinking this week!” “Thanks for coming in today. It’s good to see you!” **R**eflections: Nonjudgmentally reflecting back something that your patient has said (with an emphasis on reflecting back language “change language”). For example, “You increased your alcohol use after your mother died last year, and now you realize it may be time to cut back.” **S**ummarizing: Pull together several things someone told you. Often, reflections and affirmations are in summaries.

a For further suggestions regarding destigmatizing language in alcohol use care, please see.

https://nida.nih.gov/nidamed-medical-health-professionals/health-professions-education/words-matter-terms-to-use-avoid-when-talking-about-addiction

Responses to alcohol queries may also be influenced by stigma and shame. Alcohol use is highly stigmatized, and patients frequently feel a great deal of shame when confronted with a situation where they may have to disclose their alcohol consumption.^35^ Added to this, cirrhosis and liver disease themselves are highly stigmatized,^36,37^ which is largely because cirrhosis is closely associated in the public mind with alcohol use. Stigma and shame operate on many levels in patients with cirrhosis, including social stigma (when others avoid the patient or the patient is treated in a discriminative way due to their liver disease) or self-stigma (as when patients fear disclosing their illness or feel they must self-punish or self-alienate as a result of their liver disease).^36^ This manifests in clinics for those consuming alcohol as potentially reduced disclosure of alcohol use and lower levels of help-seeking for AUD treatment. Such high levels of stigma can also tragically prevent patients from recognizing their drinking problems or lead patients to decide that they are unworthy or undeserving of help for their alcohol use or, even, for medical care and liver transplantation, often leading to even more substance use. Frequently, the pressures of the medical encounter, particularly when a patient is being evaluated for liver transplantation, place enormous stress on the patient and, with consequences for a slip or relapse so high, may incentivize a lack of candor around alcohol consumption.

Counteracting the stigma associated with alcohol use starts by compassionately and kindly addressing alcohol use as a normal and expected part of the hepatology encounter, a health behavior similar to others discussed with patients, which also contributes to and causes other medical problems (Table 1). Compassionate and respectful inquiry forms a major basis of motivational interviewing (MI), a patient-centered interviewing approach to behavior change that is particularly helpful in those who may be ambivalent about change.^34^ Stigma related to addiction has persisted for many reasons, but one major reason is the resistance to consider addiction a true disease, as it is currently conceptualized in addiction research.^38^ Alcohol use alters brain circuitry responsible for stress, mood, and reward,^39,40^ and susceptibility to addictive substance use arises for a variety of reasons outside the control of the individual, including genetic susceptibility, social and environmental exposures, the frequent co-occurrence of other mood disorders, and traumatic life experiences, to name a few.^38^

### AUDIT-C

A helpful aid to historical questioning of alcohol consumption is the use of validated alcohol use questionnaires. The Alcohol Use Disorders Identification Test (AUDIT) is a validated tool to screen for problem alcohol consumption that is recommended for use.^9^ It can be programmed in many electronic health records and pushed out to patients ahead of their appointments, whether in person or virtual, allowing for efficient querying of alcohol consumption during the clinical encounter. The AUDIT has 2 versions, a shorter 3-question version called the AUDIT-C and a longer 10-question version (AUDIT).^41^ For the shorter AUDIT-C, values of 3 or more in women and 4 or more in men are considered positive and suggestive of heavy drinking and need for intervention, while, for the 10-question AUDIT, values of 8–14 suggest hazardous or harmful consumption and values of 15 or more may suggest a moderate to severe AUD.

### Alcohol biomarkers

Alcohol biomarkers are important adjuncts to the history and screening questions about alcohol use and are increasingly used in hepatology care, as recommended by guidelines.^9^ Alcohol biomarkers are urine, hair, and blood moieties that indicate alcohol use. They come in 2 broad categories: indirect and direct. Indirect biomarkers (ie, AST:ALT ratios, elevated gamma glutamyl-transferase levels, and high mean corpuscular volume) reflect the toxic effects of alcohol on organs, tissues, or body chemistry and do not directly represent alcohol metabolites. By contrast, direct alcohol biomarkers are direct products of ethanol metabolism. These include the blood alcohol level, urine ethyl glucuronide, urine ethyl sulfate, hair ethyl glucuronide, and phosphatidylethanol (PETH). The duration of drinking detected varies by biomarker, ranging from ~12 hours for blood alcohol levels to several months for hair ethyl glucuronide (Table 2), and assay availability also varies by the health system. Urine and hair ethyl glucuronide, urine ethyl sulfate, and phosphatidylethanol have been validated in patients with liver disease, including cirrhosis patients and those both pre-transplant and post-transplant, with varying degrees of sensitivity and specificity (Table 2). Discordance rates for alcohol biomarkers with reported abstinence by history have been estimated between 10% and 30%.^42^ Principles for using alcohol biomarkers include notifying patients that you will be checking biomarkers, normalizing their use as a regular part of clinical care like other objective markers of health behaviors (eg, hemoglobin A1c, body weight, and serum drug levels), and reassuring patients that use of biomarkers is not intended to “catch” or punish patients but rather to detect slips and relapses to aid in offering appropriate care and supporting patients in re-establishing abstinence. In addition, while dose estimations may be possible with PETH,^43^ precise dose estimation with most alcohol biomarkers is difficult, so the use of biomarkers to indicate the presence or absence of alcohol use of any amount, followed by a discussion with the patient, is recommended. In addition, false positives and false negatives are found with each biomarker, making knowledge of this critical alongside querying for potential reasons for false testing when using these in the clinic, particularly for high-stakes decisions, such as transplant candidacy.^44^

**TABLE 2:** Detection time and detection accuracy in clinic use among liver disease patients for various alcohol biomarkers

Test	Source	Detection time	Sensitivity (%)	Specificity (%)	PPV (%)	NPV (%)
Ethyl glucuronide	Urine	3–5 d	76–89	93–99	64–100	86–93
Ethyl glucuronide	Hair	Months	81–100	83–98	68–95	86–100
Ethyl sulfate	Urine	3–5 d	82	86	70	93
Phosphatidylethanol	Blood	2–3 wk	98–100	66–96	85	100

Abbreviations: NPV, negative predictive value; PPV, positive predictive value.

### Diagnosing AUD

**FIGURE 2:**
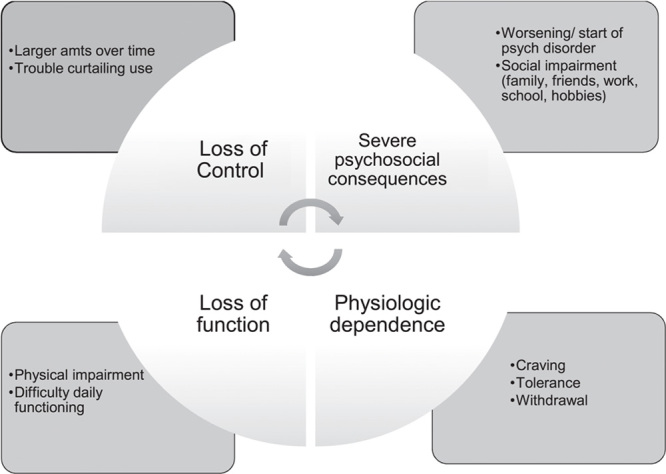
Diagnosis of alcohol use disorder, according to criteria from the Diagnostic and Statistical Manual of Mental Disorders.

Once alcohol use has been established, determining the severity of a potential alcohol use problem is important, as this can determine what alcohol treatments might be best indicated. Risky drinking is defined, as noted above, as more than 1 drink per day for women or 2 drinks per day for men. Heavy drinking is considered anything greater than 4 drinks per day for women or 5 drinks per day for men, while binge drinking consists of consuming similar amounts as heavy drinking but over a shorter time period, often 2–3 hours. AUD, by contrast, is defined not by the dose of alcohol consumed but by the consequences experienced due to alcohol consumption of whatever amount. Previously called alcohol abuse or dependence or alcohol addiction, AUD is categorized using the Diagnostic and Statistical Manual of Mental Disorders, Fifth Edition as mild, moderate, or severe based on 11 symptom criteria, which include the accumulation of negative consequences across a range of life categories (Figure 2). To diagnose AUD, the patient should be interviewed by a trained professional with expertise in addiction, such as a social worker, psychologist, or psychiatrist. However, shorter screening surveys, such as the 10-item AUDIT, discussed above, can assess the risk of an AUD using standard risk cutoff scores (≥15 may indicate moderate to severe AUD). Those who score high on the AUDIT could be referred for more formal evaluation.

## ALCOHOL USE TREATMENT

For patients with liver disease and AUD or problem alcohol use, liver and other health-related consequences can be severe. For those with ALD, especially cirrhosis or AAH, the consequences of ongoing drinking, even at low amounts, are high with increases in decompensating events, such as variceal bleeding, ascites, and HE, increased rates of HCC, higher recurrence of cirrhosis and mortality after transplantation, and higher overall liver-related mortality.^11–13,45–47^ Because of the effect of any alcohol on mortality and hepatic decompensation for those with ALD, particularly for those with cirrhosis or AAH, total abstinence is the recommended goal for all patients with ALD, who are actively drinking in any amount. However, total abstinence is often difficult for patients with ALD to achieve. Elsewhere in the AUD treatment community, harm reduction approaches are frequently used. Harm reduction seeks to move patients toward alcohol consumption goals that the patient sets and which may not include total abstinence.^48^ Harm reduction, as the name implies, seeks to reduce the harms of substance use without necessarily requiring total abstinence and individualize the approach to alcohol counseling by setting alcohol reduction or cessation goals with our patients, instead of for our patients.

### Alcohol use treatment options

#### Brief interventions

For some patients with more mild AUD symptoms, brief behavioral interventions delivered in the clinic may be beneficial. Such interventions consist of a short, 10–15 minute, in-clinic discussion with a health care provider focused on education about the harms of alcohol use and the benefits of reduction or cessation and patient-centered goal-setting and MI to help promote changes in alcohol use. Although brief interventions are largely not effective in those with more severe AUD, they can be beneficial in those with milder alcohol misuse symptoms and have been tested in several settings, including primary care, inpatient, and emergency departments.^49–52^ A meta-analysis of brief interventions, most of which were performed in primary care or emergency department settings, found that, compared with minimal to no intervention, patients who received brief interventions consumed less alcohol (mean difference −20 g/week, CI, −29 to −12).^53^ For those with more severe alcohol misuse, referral to treatment is a critical component of the Screening, Brief Intervention, Referral to Treatment (SBIRT) model. However, interventions to increase referral to treatment have been largely ineffective, showing no increase in treatment utilization in a meta-analysis of referral interventions (RR: 1.08, 95% CI, 0.92−1.28).^54,55^

#### Motivational interviewing

MI is a patient-centered communication paradigm helpful in working with less motivated and ambivalent patients toward behavior change. More than a technique, MI is a style and complete approach to patient care that involves a “collaborative conversation style for strengthening a person’s own motivation and commitment to change.”^34^ The style of MI is to ask, prompt, and guide patients, rather than force or paternalistically direct, and to develop their own internal reasons and motivation for change. Motivational enhancement therapy is a more structured way of providing MI during psychotherapy. A large body of literature, including numerous systematic reviews and meta-analyses, has shown the effectiveness of MI for alcohol reduction and cessation,^56–58^ including some studies showing effect among those with ALD and other liver diseases.^59^ One study of integrated cognitive behavior therapy (CBT), motivational enhancement therapy (MET) (which is a standardized method of delivering MI), and comprehensive medical care in ALD patients found that the combination of CBT and MET delivered in 24–48 sessions over 2 years resulted in a 74% increase in abstinence in the intervention group compared with a 48% increase in the control group (*p* = 0.02).^60^ Another recent randomized pragmatic trial evaluated the effectiveness of an SBIRT intervention that incorporated MI delivered by the medical provider as a stand-alone treatment versus SBIRT plus formal alcohol treatment (a combination of CBT, MET, and substance abuse treatment delivered by an integrated mental health professional) in a group of hepatitis C patients with alcohol use.^61^ In this trial, patients in both arms had significant increases in abstinence at 6 months, with no difference between treatment arms (SBIRT-only: 20.5%; SBIRT + alcohol treatment: 23.3%; and the coefficient for intervention effect: 0.65, 95% CI, −0.65, 1.96). In the United States, training for MI is available in many forms including through the Motivational Interviewing Network of Trainers (https://motivationalinterviewing.org) and by means of online courses and educational books.

#### Other therapeutic modalities

A wide variety of other therapeutic modalities have been shown to be effective in assisting patients in reducing or stopping alcohol use. In addition to inpatient alcohol use treatment (which often combines many of the following modalities into an inpatient, residential setting), these include CBT, 12-step facilitation, contingency management, couples and family therapy, mindfulness-based relapse prevention, contingency management and community reinforcement, and social behavior and network theory (Table 3).

**TABLE 3:** Behavioral modalities for treatment of alcohol use

Behavioral therapy	Provider delivering intervention	Efficacy in ALD	Description
Screening, Brief Intervention, Referral to Treatment (SBIRT)	Any clinician	20–23% achieve abstinence w/ SBIRT	Providing screening for alcohol use, brief (usually motivationally interviewing themed) discussion on alcohol reduction, and referral to alcohol treatment where indicated.
Cognitive behavior therapy (CBT)	Trained MHSA^a^ provider	CBT/MET for 2 y: 74% increase in abstinence vs. 48% in controls	Focuses on modifying dysfunctional thoughts, emotions, and behaviors. In AUD treatment, used to identify cues and triggers for relapse, improve coping strategies, substance-refusal training, increase focus on substance-free activities.
Motivational enhancement therapy (MET)/motivational interviewing (MI)	Any clinician	CBT/MET for 2 y: 74% increase in abstinence v 48% in controls	MI and it is more structure version (MET) are widely used, evidence-based approaches for eliciting and strengthening personal motivation to change. Especially helpful for those who are ambivalent about or resistant to positive behavior change.
12-step facilitation	Trained MHSA^a^ provider	Unknown, no data	Therapy modality in which focuses on total alcohol abstinence and regular participation in 12-step/alcohol-associated anonymous meetings
Contingency management	Trained MHSA^a^ provider	Unknown, no data	Intervention based on operant conditioning where patients receive an incentive (often financial) in exchange for evidence of reduction in use or abstinence

a MHSA: mental health/substance abuse provider

Abbreviations: ALD, alcohol-associated liver disease; AUD, alcohol use disorder.

Adapted from Leggio and Mellinger.^79^

#### Community mutual aid societies

Community mutual aid societies consist of organizations of those with alcohol use problems who come together to assist and support one another in reducing alcohol or achieving and maintaining abstinence. The most well-known is Alcoholics Anonymous (AA), but other societies (such as SMART Recovery, Celebrate Recovery, and Refuge Recovery) also exist and focus on recovery from alcohol use problems using different philosophies and approaches. A recent meta-analysis of AA and 12-step facilitation (a counseling approach based on and supporting the 12-step approach to alcohol cessation) found that, among 1936 participants with alcohol use problems and compared with other treatment modalities, such as CBT or MET, AA participation plus a standardized approach to 12-step facilitation improved rates of continuous abstinence at 12 months (RR: 1.21, 95% CI, 1.03, 1.42) with a consistent effect maintained at 24 and 36 months.^62^ Although this data does not specifically include ALD patients, AA or other mutual aid societies may be especially appealing given the high availability of these groups, virtual options for meetings, and low/no cost.

#### Relapse prevention medications

Relapse prevention medications constitute an important part of the AUD treatment armamentarium (Table 4). However, because of a lack of safety data in advanced liver disease patients and unfamiliarity with prescribing among hepatologists, few patients with liver disease and AUD receive medication therapy for AUD.^69,70^ Among those who do access medication therapy for AUD, outcomes are improved, including reduced incidence and progression of ALD.^71^ In addition, those few ALD patients who do access psychosocial treatment often find that their addiction providers are uncomfortable prescribing relapse medications, some of which are metabolized by the liver, leaving these patients undertreated.^72^

**TABLE 4:** Relapse prevention medications and potential use in liver disease patients

Medication	FDA approved	Metabolism and excretion	Starting and effective dose	Liver disease considerations
Disulfiram	Yes	M: hepatic E: 70% renal	250–500 mg daily	Severe, sometimes fatal, DILI and/or acute liver failure requiring transplant. Reports of neuropathy and psychosis. Not recommended for use in liver disease.
Naltrexone	Yes	M: hepatic E: mostly renal, 2% fecal	Start: 25 mg daily Effective dose: 50 mg daily orally; 380 mg intramuscularly monthly.	Rare potential for hepatotoxicity, some documented elevations in liver enzymes but no known cases of liver failure. Can see drug and metabolite accumulation in advanced cirrhosis (Childs class B or C).^63^ Suggest oral formulation over intramuscular for those with cirrhosis. Interacts with opioids so ensure patient is not on narcotics before starting. Meta-analysis in AUD patients without liver disease showed moderate efficacy.
Acamprosate	Yes	M: no hepatic E: renal	Start: 333 mg three times daily Effective dose: 666 mg orally 3 times daily	No evidence of hepatotoxicity. Meta-analysis in AUD patients without known liver disease showed moderate efficacy.
Gabapentin	No	M: not hepatic E: 75% renal, 25% fecal	Start: no clear starting dose recommended Effective dose: 900–1800 mg 3 times daily	No hepatotoxicity. Theoretical abuse potential. Use with caution for those with HE. Dose reduce in renal failure. For those with chronic kidney disease, lower doses may be as effective.
Baclofen	No	M: limited hepatic E: renal	Start: 5 mg 3 times daily for 3 days, then increase to target dose by 5 mg every 3 day increments Effective dose: 10–20 mg 3 times daily	No evidence for direct hepatotoxicity but may precipitate HE. Has been tested in randomized trials in ALD patients ^64–66^ and other observational trials.^67,68^ Dose reduce in renal failure and avoid administering in end-stage renal disease.
Topiramate	No	M: limited hepatic E: renal	*Start:* 25–50 mg daily. Increase in 25–50 mg increments weekly to effective dose goal. *Effective dose:* 300 mg daily	No evidence for hepatotoxicity but could affect liver function. Could worsen/confound hepatic encephalopathy. Dose reductions in hepatic and renal impairment. Effective dose needed may be lower in those with more advanced liver or kidney disease.

Abbreviations: ALD, alcohol-associated liver disease; AUD, alcohol use disorder; FDA, Food and Drug Administration.

#### Disulfiram

Disulfiram, or Antabuse, is a Food and Drug Administration (FDA)-approved medication for alcohol relapse prevention. Disulfiram can be, however, highly hepatotoxic and has been linked to instances of liver failure, need for transplant, and death. It should not be used in patients with liver disease.

#### Acamprosate

Introduced in Europe in 1989, acamprosate is an FDA-approved medication for alcohol relapse prevention, which works by modulating the NMDA receptor, possibly as a partial coagonist.^73^ Acamprosate has no hepatic metabolism and no reported hepatotoxicity. It is renally metabolized, so the dose must be reduced if the glomerular filtration rate is <60, and administration should be avoided if <30. Acamprosate has been tested widely in multiple studies of patients with AUD but has never been formally tested in liver disease patients exclusively. One of the largest studies of medication-assisted therapy for AUD, the COMBINE study,^74^ evaluated the impact of 16 weeks of naltrexone or acamprosate with or without combined behavioral intervention and found that, while naltrexone was effective in reducing alcohol use at a dose of 100 mg daily by mouth daily (HR for return to heavy drinking: 0.78 (95% CI, 0.63, 0.97), acamprosate, at a dose of 1000 mg 3 times daily, was not (HR: 0.93 (95% CI, 0.75, 1.16). However, other meta-analyses have found that acamprosate is effective at promoting alcohol reduction and abstinence.^75,76^ In 1 meta-analysis, acamprosate had a number-needed-to-treat (NNT) for return to any drinking of 12 (8–26) at a dose of 666 mg 3 times daily.^75^

#### Naltrexone

Naltrexone is a potent competitive mu-opioid receptor antagonist that has been approved by the FDA for alcohol relapse prevention. Like acamprosate, it has been widely tested in the general AUD population but has not had any formal randomized trials in patients with liver disease. Naltrexone is metabolized by the liver, so concerns have been raised about hepatotoxicity. A small pharmacokinetic study performed in 18 patients with no cirrhosis, compensated cirrhosis (Childs A-B), and decompensated (Childs C) cirrhosis showed that elimination half-life was similar between groups (with slight increase in decompensated cirrhosis), but that the primary drug, naltrexone, and metabolite, 6β-natrexol, responsible for most of the pharmacologic effects, were both more elevated in cirrhosis patients.^63^ Elevated liver enzymes have been reported with the use of naltrexone, but there have been no reported cases of liver failure or liver-related death due to its use. Naltrexone does seem to be effective in several meta-analyses in the general AUD population. In one large meta-analysis, naltrexone had an NNT of 20 (11–500) for return to any drinking and 12 (8–26) for return to heavy drinking at a dose of 50 mg orally per day. In the COMBINE study,^74^ naltrexone for 16 weeks was found to be effective at promoting abstinence, with or without combined behavioral interventions (HR for return to heavy drinking: 0.78 (95% CI, 0.63, 0.97). Because of concerns about hepatotoxicity, the use of naltrexone has been limited, but a recent retrospective study in liver disease patients suggests that this concern may be overstated.^77^ However, a risk/benefit analysis should be undertaken tailored to each patient and considering the severity of AUD, the need for anticraving agents, and the stage of liver disease.

#### Baclofen

Unlike most other relapse prevention medications, baclofen, a GABA-B receptor agonist, has been tested in patients with advanced ALD, including those with cirrhosis. It is largely metabolized in the kidney, not the liver, so has no reported direct hepatotoxicity. In a small randomized controlled trial of patients with Childs A, B, and C cirrhosis in Italy,^64^ those randomized to baclofen at a dose of 10 mg 3 times daily had more total abstinence [30/42 (71%) versus 12/42 (29%), *p* = 0.02] at 12 weeks compared with controls. The study was limited by exclusion criteria, which included HE, the presence of pharmacologic treatment for other mental illnesses, and other substance use (other than tobacco), limitations that may reduce generalizability. Several other observational, uncontrolled studies of baclofen in ALD patients have also been published, which have shown reductions in alcohol consumption and an increase in total abstinence in patients with all stages of ALD, including cirrhosis.^67,68,78^ However, a randomized trial of baclofen 30 mg daily for 12 weeks in veterans with chronic hepatitis C and active alcohol use showed no difference between placebo and baclofen on abstinence rates.^65^ A more recent randomized trial, the BacALD trial, examined the effects of baclofen at either 10 mg 3 times daily or 25 mg 3 times daily for 12 weeks in a cohort of patients with and without ALD.^66^ Abstinence rates were similar between the baclofen 30- and 75-mg groups (21% and 23% respectively, *p* = 0.15) with improvement over placebo (10%). Critically, more patients experienced adverse events in the baclofen 75-mg group, with 51% reporting significant sedation or drowsiness. For those with cirrhosis, precipitation of HE is a concern, and thus, baclofen should be used cautiously in those with cirrhosis and any history of HE. It should not be used in those with ongoing or poorly controlled HE. Importantly, all of these studies, including the randomized baclofen trials, included some form of behavioral therapy alongside baclofen administration. Also, given their metabolism and excretion by the kidney, baclofen should be avoided in those with end-stage renal disease.

#### Other medications

Other non-FDA–approved medications for potential use include topiramate, gabapentin, and varenicline. Topiramate is an antiepileptic medication, which acts by decreasing dopaminergic activity and enhancing GABA action, and is FDA-approved for the treatment of epilepsy, migraines, and (when combined with phentermine) obesity.^79^ It is not extensively metabolized and is cleared renally, but clearance can be reduced by hepatic insufficiency. Topiramate has been shown in several clinical trials to reduce alcohol consumption and achieve alcohol abstinence, but caution is warranted, particularly in those with any degree of hepatic encephalopathy, as some patients have reported side effects, including mental status changes.^80–82^ Gabapentin is a well-known, widely used medication for neuropathy and nonopioid pain relief that also has evidence in the general AUD population for some benefits in promoting abstinence, improving alcohol withdrawal symptoms, and reducing cravings.^83–86^ Gabapentin modulates GABA and glutamate antagonism, and has no known hepatotoxicity. It is renally cleared and, in advanced liver disease patients with varying renal function, caution must be used given the risk of altered mental status. Any medication that sedates a patient should raise caution for causing or worsening HE and must be carefully weighed against its benefits and followed closely. Gabapentin may also be particularly helpful in reducing alcohol use in those with alcohol withdrawal symptoms and has been shown in retrospective studies to reduce inpatient benzodiazepine use for alcohol withdrawal and shorten the length of stay in those admitted for alcohol intoxication and withdrawal.^84,87,88^ Consideration can be given to starting this medication inpatient for patients experiencing mild withdrawal and then continuing it into the outpatient setting for additional anticraving benefits. Varenicline, a nicotinic acetylcholine receptor partial agonist, is FDA-approved for tobacco cessation and has some evidence to suggest that those with both AUD and tobacco cessation saw improved alcohol-associated outcomes as well when on varenicline.^89–92^ Varenicline is less well-studied for AUD than gabapentin or topiramate, however, and caution in use is warranted for those with renal disease. For all relapse medication use in liver disease, a thorough understanding of metabolism and side effects is necessary to appropriately weigh risks and benefits before prescribing.

## INTEGRATED CARE FOR ALCOHOL USE AND LIVER DISEASE

Patients with liver disease and comorbid AUD frequently do not receive adequate treatment for their alcohol use problems, in part because of discomfort among hepatologists and addiction specialists regarding prescribing relapse prevention medications in liver disease patients and due to inadequate referrals to AUD treatment.^69,70,72^ However, improving outcomes for patients with AUD and liver disease requires integrating alcohol use and liver disease treatment across multiple domains of care.^93^ Integrated care is a well-established idea that focuses on care, which is team-driven, population-focused, measurement-guided, evidence-based, and improves quality.^93^ For those with the most advanced liver disease (cirrhosis and/or alcoholic hepatitis) and AUD, integrated multidisciplinary clinics and inpatient consult teams have shown promise in improving outcomes.^94,95^ Integrated care is, however, a spectrum, ranging from incorporating routine alcohol questioning with AUDIT or other surveys all the way to fully integrated, colocated ALD clinics. Local resources for creating a fully integrated ALD clinic may be limited, but no matter where a provider practices, integrating alcohol use care with liver care is feasible at some level. Although achieving improved outcomes for patients with AUD and liver disease, integrated care models also require strong interprofessional relationships based on mutual respect, shared knowledge and goals, and strong communication practices focused on frequent, timely, accurate, and problem-solving communication.^93,96–101^ Although a full discussion of how to implement integrated care for AUD and liver disease is beyond the scope of this article, additional, more detailed information on the implementation of integrated care can be found in the reference list.^93,94,96–99^ Nonetheless, clinicians should integrate alcohol use and liver disease as much as possible to improve outcomes for patients.

## CONCLUSIONS

Management of AUD and liver disease requires integrating alcohol use detection, diagnosis of severity of AUD, and direction to treatment, which often means referral to higher levels of care, into the standard hepatology practice. At the bedside, the concerned liver clinician can provide compassionate alcohol detection (through interviews, surveys, and alcohol biomarkers) in the context of a MI approach that gently and kindly centers the patient’s concerns and respectfully recognizes the ambivalence that patients may feel when asked to reduce or stop alcohol use. The use of relapse prevention medications is recommended and can be a powerful adjunct to behavioral therapies to help patients stop alcohol use. Clinicians should feel empowered to start such medications and monitor for effectiveness and side effects. Only when AUD care is integrated into liver care, even in the most foundational of ways, such as routinely asking about alcohol use and utilizing the AUDIT screen, we can begin to turn the tide on alcohol’s impact on liver disease.

## Supplementary Material
