# Supplementary material for: Management of alcohol use disorder in patients with chronic liver disease
Source: Hepatol Commun. 2023 Jun 14;7(7):e00145. doi: 10.1097/HC9.0000000000000145 (PMC10270526; doi:10.1097/HC9.0000000000000145)
Supplement: Supplementary file 1 [file hc9-7-e00145-s001.docx]

**Supplementary Table 1.** Comparison of sociodemographic and clinical characteristics among waitlist additions and liver transplant recipients in the United States prior to and during the COVID-19 pandemic.

|  | **Liver Transplant Recipients** | | |
| --- | --- | --- | --- |
|  | **Pre-COVID Era** | **COVID Era** |  |
|  | December 1, 2017 – February 28, 2019  N = 6209 | March 1, 2020 – June 1, 2021  N = 7620 | *P* |
| **Median age, [IQR]** | 57 [15] | 56 [17] | <0.001 |
| **Age distribution, n (%)** |  |  | <0.001 |
| 18-34 | 457 (7.4) | 621 (8.1) |  |
| 35-49 | 1272 (20.5) | 1909 (25.1) |  |
| 50-64 | 3219 (51.8) | 3594 (47.2) |  |
| > 65 | 1261 (20.3) | 1496 (19.6) |  |
| **Gender, n (%)** |  |  | 0.952 |
| Female | 2296 (37.0) | 2814 (36.9) |  |
| Male | 3913 (63.0) | 4806 (63.1) |  |
| **Ethnicity/Race, n (%)** |  |  | 0.682 |
| Caucasian | 4438 (71.5) | 5450 (71.5) |  |
| Black | 487 (7.8) | 555 (7.3) |  |
| Hispanic | 933 (15.0) | 1190 (15.6) |  |
| Asian | 240 (3.9) | 294 (3.9) |  |
| Other | 111 (1.8) | 131 (1.7) |  |
| **Etiology of liver disease, n (%)** |  |  |  |
| Chronic HCV | 1198 (19.3) | 955 (12.5) | <0.001 |
| ALD | 2305 (37.1) | 3604 (47.3) | <0.001 |
| NASH | 1328 (21.4) | 1693 (22.2) | 0.240 |
| **Median serum MELD-Na score at listing, [IQR]** | 23 [16] | 26 [15] | <0.001 |
| **MELD-Na score at listing** |  |  | <0.001 |
| < 20 | 2351 (37.9) | 2209 (29.0) |  |
| 20-24 | 1032 (16.6) | 1223 (16.0) |  |
| 25-29 | 875 (14.1) | 1275 (16.7) |  |
| 30-34 | 826 (13.3) | 1272 (16.7) |  |
| > 35 | 1125 (18.1) | 1641 (21.5) |  |
| **Median serum MELD-Na score at transplant, [IQR]** | 26 [17] | 28 [14] | <0.001 |
| **MELD-Na score at transplant, n (%)** |  |  | <0.001 |
| < 20 | 1960 (31.6%) | 1818 (23.9%) |  |
| 20-24 | 903 (14.5%) | 1156 (15.2%) |  |
| 25-29 | 930 (15.0%) | 1362 (17.9%) |  |
| 30-34 | 877 (14.1%) | 1431 (18.8%) |  |
| > 35 | 1539 (24.8%) | 1853 (24.3%) |  |
| **Hepatic decompensation at transplant, n (%)** |  |  |  |
| Moderate or severe ascites | 2266 (36.5) | 3133 (41.1) | <0.001 |
| Spontaneous bacterial peritonitis | 688 (11.1) | 907 (11.9) | 0.132 |
| Severe hepatic encephalopathy | 994 (16.0) | 1257 (16.5) | 0.440 |
| Hepatocellular carcinoma | 1442 (23.2) | 1212 (15.9) | <0.001 |
| **Hemodialysis at transplant, n (%)** | 1284 (20.7) | 1665 (21.9) | 0.095 |
| **On mechanical ventilation or life support at transplant, n (%)** | 818 (13.2) | 785 (10.3) | <0.001 |

Abbreviations: ALD, alcohol-associated liver disease; HCV, hepatitis C virus; MELD-Na, Model for End-Stage Liver Disease-Sodium; NASH, nonalcoholic steatohepatitis; SAH, severe acute alcohol-associated hepatitis
